# Three-Dimensional Printing of a Hybrid Bioceramic and Biopolymer Porous Scaffold for Promoting Bone Regeneration Potential

**DOI:** 10.3390/ma15051971

**Published:** 2022-03-07

**Authors:** Kuo-Sheng Hung, May-Show Chen, Wen-Chien Lan, Yung-Chieh Cho, Takashi Saito, Bai-Hung Huang, Hsin-Yu Tsai, Chia-Chien Hsieh, Keng-Liang Ou, Hung-Yang Lin

**Affiliations:** 1 Graduate Institute of Injury Prevention and Control, College of Public Health, Taipei Medical University, Taipei 110, Taiwan; kshung@tmu.edu.tw; 2 Department of Neurosurgery, Taipei Medical University-Wan Fang Hospital, Taipei 116, Taiwan; 3 School of Dentistry, College of Oral Medicine, Taipei Medical University, Taipei 110, Taiwan; maychen@tmu.edu.tw (M.-S.C.); d204106003@tmu.edu.tw (Y.-C.C.); 4 Division of Prosthodontics, Department of Dentistry, Taipei Medical University Hospital, Taipei 110, Taiwan; 5 Department of Oral Hygiene Care, Ching Kuo Institute of Management and Health, Keelung 203, Taiwan; jameslan@ems.cku.edu.tw; 6 Biomedical Technology R & D Center, China Medical University, Taichung 404, Taiwan; u109312001@cmu.edu.tw; 7 Division of Clinical Cariology and Endodontology, Department of Oral Rehabilitation, School of Dentistry, Health Sciences University of Hokkaido, Ishikari 061-0293, Japan; t-saito@hoku-iryo-u.ac.jp (T.S.); m225098012@tmu.edu.tw (H.-Y.T.); 8 Graduate Institute of Dental Science, College of Dentistry, China Medical University, Taichung 404, Taiwan; 9 Graduate Institute of Biomedical Optomechatronics, College of Biomedical Engineering, Taipei Medical University, Taipei 110, Taiwan; d204095002@tmu.edu.tw; 10 3D Global Biotech Inc. (Spin-Off Company from Taipei Medical University), New Taipei City 221, Taiwan; 11 Taiwan Society of Blood Biomaterials, New Taipei City 221, Taiwan; 12 Department of Dentistry, Taipei Medical University-Shuang Ho Hospital, New Taipei City 235, Taiwan; 13 Department of Dentistry, Fu Jen Catholic University Hospital, Fu Jen Catholic University, New Taipei City 242, Taiwan

**Keywords:** 3D printing, bone regeneration, biocompatibility, tricalcium phosphate, pluronic F127

## Abstract

In this study, we proposed a three-dimensional (3D) printed porous (termed as 3DPP) scaffold composed of bioceramic (beta-tricalcium phosphate (β-TCP)) and thermoreversible biopolymer (pluronic F-127 (PF127)) that may provide bone tissue ingrowth and loading support for bone defect treatment. The investigated scaffolds were printed in three different ranges of pore sizes for comparison (3DPP-1: 150–200 μm, 3DPP-2: 250–300 μm, and 3DPP-3: 300–350 μm). The material properties and biocompatibility of the 3DPP scaffolds were characterized using scanning electron microscopy, X-ray diffractometry, contact angle goniometry, compression testing, and cell viability assay. In addition, micro-computed tomography was applied to investigate bone regeneration behavior of the 3DPP scaffolds in the mini-pig model. Analytical results showed that the 3DPP scaffolds exhibited well-defined porosity, excellent microstructural interconnectivity, and acceptable wettability (θ < 90°). Among all groups, the 3DPP-1 possessed a significantly highest compressive force 273 ± 20.8 Kgf (* *p* < 0.05). In vitro experiment results also revealed good cell viability and cell attachment behavior in all 3DPP scaffolds. Furthermore, the 3DPP-3 scaffold showed a significantly higher percentage of bone formation volume than the 3DPP-1 scaffold at week 8 (* *p* < 0.05) and week 12 (* *p* < 0.05). Hence, the 3DPP scaffold composed of β-TCP and F-127 is a promising candidate to promote bone tissue ingrowth into the porous scaffold with decent biocompatibility. This scaffold particularly fabricated with a pore size of around 350 μm (i.e., 3DPP-3 scaffold) can provide proper loading support and promote bone regeneration in bone defects when applied in dental and orthopedic fields.

## 1. Introduction

Nowadays, various synthetic bone graft materials have been used to fill the gap of large bone defects and promote bone regeneration [1,2,3]. Among these synthetic materials, beta-tricalcium phosphate (β-TCP) is of great interest owing to its biocompatibility and bioactivity similar to the natural bone structures [4,5,6]. The β-TCP offers a great balance between absorption, degradation, and the formation of new bones [5,7,8]. This material can be used directly for bone replacement or in combination with other ceramic materials for biomedical applications [9,10]. Although β-TCP is considered an optimal material for bone tissue engineering, it is brittle in shear and tension due to its mechanical characteristics [8,11,12]. In light of that, to mitigate the limitation of a single material, bone graft scaffolds fabricated from the mixture between synthetic biodegradable polymers and osteoconductive ceramic particles have become a forefront topic in the field of biological material and tissue engineering [6,13].

The incorporation of different classes of materials can particularly improve the geometric specificity of a three-dimensional (3D) printing scaffold [14,15]. When engineering hard tissues like bone, porous structures integrated with high mechanical resistance are extremely advisable [3,8,16,17]. Among all biomaterials used in bone tissue engineering, hydrogels are the most promising ones since they are suitable to be used for targeted different properties and specific applications [2,18]. In recent years, one type of hydrogel, pluronic F-127 (PF127) has attracted particular interest [19]. The PF127 is a typical thermoreversible polymer with unique micellar properties and gelation behavior [20]. This material is liquid at 4 °C and becomes a gel at 37 °C within 5 min [18]. Over the past years, this polymer has been extensively used as a carrier for drug and gene delivery and inhibition of tissue adhesion [19,20,21]. Recent advances in the use of PF127 were reported for bone tissue engineering [19]. This material is known to have good printing properties and is structurally robust but weak cell compatibility that can prevent their single use as bone substitutes [14,22]. In order to utterly exploit the 3D printing potential, it is prominent to develop appropriate material combinations to generate an ideal bone scaffold that can convoy the ingrowth of vascular network as well as offering structural support throughout bone remodeling [11,16], while previous studies have indicated that a pore size between approximately 200 and 350 µm is ideal for vascularization potential and osteoblast proliferation [22,23].

Therefore, the present study aimed to combine the desirable effect from β-TCP and PF127 through the generation of a 3D printed porous scaffold containing both of these materials. Since it is fabricated via the 3D printing technic, the proposed porous scaffold could be customized and designed according to the defect area. We expected that the hybrid β-TCP and PF127 scaffolds with pore sizes between approximately 200 and 350 µm would be favorable to provide bone tissue ingrowth and loading support for bone defects treatment. To test this hypothesis, we investigated the property of the generated 3DPP scaffolds by morphological analysis, wettability measurement, compressive strength evaluation, and cell culture assay. Moreover, in vivo mini-pig experiments were conducted to analyze bone regeneration of the 3DPP scaffolds for future clinical applications.

## 2. Materials and Methods

### 2.1. Materials Preparation

In order to formulate the β-TCP powder, the precursor with a molar ratio of 1 mol calcium carbonate (CaCO_3_, purity ≥ 98.5%, Honeywell Fluka^TM^ Inc., Seelze, Germany): 2 mol calcium phosphate dibasic anhydrous (CaHPO_4_, purity ≥ 99%, Honeywell Fluka^TM^ Inc., Seelze, Germany) was added in 200 mL of deionized water. Subsequently, the mixture solution (pH~8.5) was stirred with a magnetic stirring bar for 1 h at a temperature of 25 °C. Hereafter, the mixture was dried for 8 h at 40 °C and then sintered at 1100 °C for 1 h based on the following Equation (1):

CaCO_3_ + 2 CaHPO_4_ → Ca_3_ (PO_4_)_2_ + H_2_O + CO_2_ (1)

The acquired β-TCP powder was manually ground and sieved using a 250-mesh sieve. Afterward, the sieved β-TCP powder was mixed with PF127 hydrogel (Sigma 0.709 mmol, Taipei, Taiwan) in a weight ratio of 3:2 and stirred at 4 °C for 30 min. Finally, the mixture of β-TCP and PF127 was moved to the 10 mL printing syringe and kept at 37 °C as gel ink.

### 2.2. 3D Printing Fabrication

A self-assembled 3D bioprinter with an extruded syringe dispenser system was utilized to print three different ranges of pore sizes scaffolds (3DPP-1: 150–200 μm, 3DPP-2: 250–300 μm, and 3DPP-3: 300–350 μm). The protocol to generate a 3D porous construct pattern was designed by the CAD software (Dassault Systèmes SolidWorks Corporation, 2014 version, Waltham, MA, USA). Before printing, the glass slide substrate was placed on the temperature-controllable platform and elevated temperature to 37 °C. Subsequently, the 3DPP scaffolds were printed layer by layer as a cylindrical shape with a 12 mm diameter and a thickness of 3 mm (according to the design patterns) on the substrate under the same printing parameters including feed rate (6.0 mm/s), printing pressure (3.0 bar), and pinhead (diameter: 0.51 mm). Lastly, the printed scaffolds were dried thoroughly in an electronic dry oven. All printing procedures, microstructural and in vitro properties of the generated scaffolds were analyzed.

### 2.3. Properties Analysis

Topography characteristics of the fabricated 3DPP scaffolds were observed through a field-emission scanning electron microscope (FE-SEM; JEOL-6500F, Tokyo, Japan). The compositions of β-TCP inside the fabricated scaffold were evaluated using an INCA energy-dispersive X-ray spectrometer (EDS; Oxford Instruments, Abingdon, UK) at an accelerating voltage of 20 kV. Moreover, phase identification and crystallinity were analyzed by X-ray diffractometer (XRD; Rigaku 2200, Tokyo, Japan) with CuKα1 radiation performed at 250 mA and 50 kV. The corresponding peaks of the XRD pattern were examined according to the Joint Committee on Powder Diffraction Standards (JCPDS) database.

### 2.4. Wettability Evaluation

A sessile drop procedure was performed using a GBX DGD-DI contact angle goniometer (Romans sur Isère, France) to assess the wettability of fabricated 3DPP scaffolds. Cylindrical constructs of 3DPP samples were prepared and deionized water drops (a droplet with a volume of ~5 μL) were dripped on the surface of 3DPP-1, 3DPP-2, and 3DPP-3, respectively with five times (*n* = 5) repetitions. A line tangent to the dropped-deionized water and the surface of 3DPP scaffold samples was adopted as the contact angle. Hereafter, an average contact angle for each sample was recorded.

### 2.5. Mechanical Testing

A compression force analysis was carried out in the fabricated 3DPP scaffolds by means of an LF Plus digital testing machine (Lloyd Instruments Ltd., Hampshire, UK) under an initial strain rate of 3.5 × 10^−4^/s and temperature of 25 °C. An average of three (*n* = 3) tests per sample were performed in the testing.

### 2.6. Cytotoxicity Assessment

The osteoblast-like cell line (MG-63, ATCC-CRL1427, The Bioresource Collection and Research Center, Hsinchu, Taiwan) was used in this study. Cells were expanded in Eagle’s Minimum Essential Medium (MEM, Gibco, Thermo Fisher scientific, Waltham, MA, USA) supplemented with 10% fetal bovine serum (FBS), 100 IU/mL penicillin, 100 µg/mL streptomycin, and 1% glutamine at 37 °C with an atmosphere of 5% CO_2_ and 95% air. To observe cell morphology and adhesion behavior on the 3DPP scaffolds, the cells were seeded into each 3DPP-1, 3DPP-2, and 3DPP-3 scaffold. Prior to cell seeding, the scaffolds were sealed in the sterile pouch and sterilized via ethylene oxide (3M 8XL, 3M, Saint Paul, MN, USA). The sterilized 3DPP scaffolds (*n* = 5) were then cultured with MG-63 cell suspension at a density of 5 × 10^4^ cells/well and maintained for one day in an incubator (37 °C, 5% CO_2_). After 1 day of incubation, 50 mL of 3-[4,5-dimethylthiazol-2-yl]-2,5-diphenyltetrazolium bromide (MTT, Sigma, Taipei, Taiwan) solution was pipetted into each culture well and the culture plate was incubated for 4 h to form the formazan solvent precipitates. The precipitated formazan was solubilized in 150 mL of dimethyl sulfoxide, and absorbance was measured through an Epoch microplate reader at 595 nm (BioTek Instruments Inc., Winooski, VT, USA). The cell viability (%) in the short-term culturing experiment was adopted to assess the material’s acute cytotoxicity response according to ISO 10993-5 specification.

### 2.7. Cell Morphology Observation

The morphology of MG-63 cells was analyzed after 3 days of culture. The adhered MG-63 cells were washed with PBS, placed in a fixative consisting of 2.5% glutaraldehyde in 0.1 M sodium cacodylate buffer for 1 h in 4 °C, rinsed in deionized water, and dehydrate in serial of ethanol solutions for 15 min each concentration. Afterwards, dehydrated samples were soaked in hexamethyldisilazine, sputter coated with platinum, and observed with JEOL-6500F FE-SEM at 20 kV under different magnifications.

### 2.8. Animal Model and Implantation Procedure

The in vivo pilot study was conducted in nine mini-pigs that were purchased from the National Laboratory Animal Center (Taipei, Taiwan). The animal use protocol of this study has been reviewed by the institutional animal care and use committee for Taipei medical university with an approval number of LAC-2014-0050. This prospective controlled study was conducted following ISO 10993-6:2016 standard regarding the biological evaluation of medical devices—Part 6: Tests for local effects after implantation. The surgical implantation procedure was done under sterile and aseptic conditions. Zoletil 50 and Xylazine were used for general anesthesia and maintained with Isoflurane through inhalation. For the implant placement, a 5 cm to 8 cm incision was made, after that, the cortex and the muscular layer were removed until the white bone can be seen. Each bone defect with 12 mm of diameter and 3 mm of thickness was made via a trephine ring saw. Afterward, the 3DPP-1, 3DPP-2, and 3DPP-3 scaffolds were randomly implanted in the left and right side of the lateral condyle, subsequently, the wound defect was closed and sutured. The bone formation and healing process of the created defect were analyzed at 4, 8, and 12 weeks after implantation. 

### 2.9. Micro-Computed Tomographic (CT) Investigation

Before the experiment, the animals were anesthetized for preoperative micro-CT images. The mini pig connected to the gas anesthesia device to maintain the stable animal physiological conditions after surgery. The Bruker Skyscan 1176 micro-CT scanner (Kontich, Belgium) was utilized to scan the implanted scaffolds at a high resolution of 18 μm. The micro-CT scanning was set at 400 ms integration time, 300 μA current, and 80 kV voltage facilitated with aluminum filter and copper. For section reconstructions, GPU-based scanner software (NRecon, Kontich, Belgium) was employed. The region of interest (ROI) was determined at 1 mm from the edge of the lateral condyle to include all implanted scaffolds. For calculation of new bone formation, a volume rendering software CTVox (Kontich, Belgium) was utilized. New bone volume is presented as a percentage of tissue volume (bone volume (BV)/total volume (TV), %). An average of three readings (*n* = 3) per sample was calculated in the experiment.

### 2.10. Statistical Analysis

Data were analyzed through SPSS statistic software (Version 19.0., SPSS Inc., Chicago, IL, USA). The difference between multiple groups was determined by one-way analysis of variance followed by Tukey’s HSD post hoc test. Statistical significance was considered with *p* values ≤ 0.05.

## 3. Results

### 3.1. Morphology and Microstructure of the Investigated 3DPP Scaffolds

**Figure 1:**
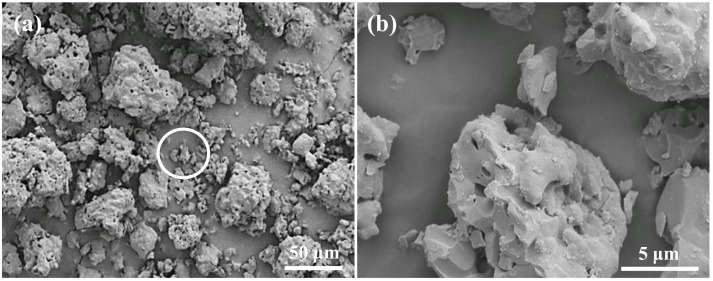
FE-SEM micrographs of (**a**) the synthesized β-TCP particles and (**b**) a higher magnification image taken from the white circle area in (**a**).

**Figure 2:**
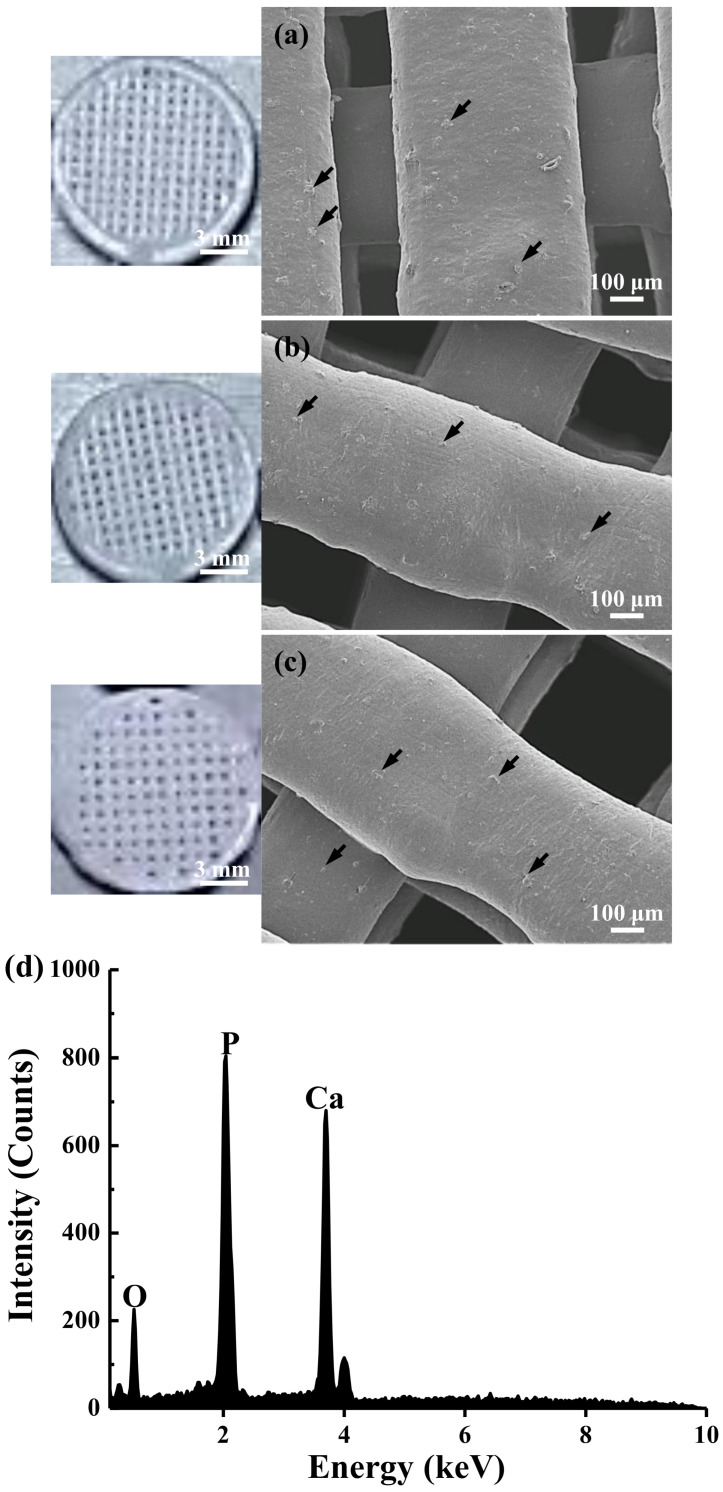
Morphology and chemical compositions of the investigated scaffolds: (**a**) 3DPP-1, (**b**) 3DPP-2, (**c**) 3DPP-3, and (**d**) an EDS spectrum taken from the surface of the printed 3DPP-1 scaffold. The FE-SEM observation and EDS analysis confirmed the presence of β-TCP particles (as pointed by arrows) in the printed lines.

**Figure 3:**
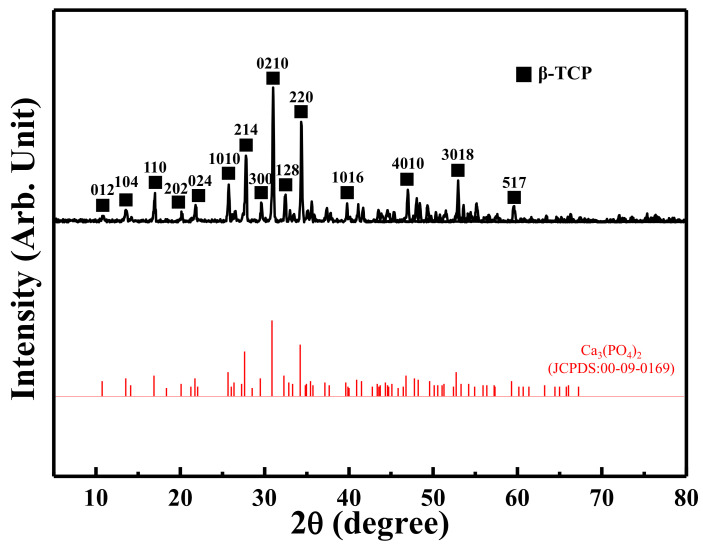
XRD pattern taken from the investigated 3DPP-1 scaffold.

As shown in Figure 1a,b, the particle size of β-TCP material was approximately 10~50 µm. Analysis of the structural interval of the fabricated scaffolds revealed that the average pore sizes of the 3DPP-1, 3DPP-2, and 3DPP-3 scaffolds were around 200 μm, 270 μm, and 350 μm, respectively (Figure 2a–c). It was also found that many β-TCP particles (as pointed by arrows) were localized in printed lines. Overall, the FE-SEM analysis indicated that the morphology of generated 3DPP scaffolds has well-manufactured and interconnected porous structures. Figure 2d depicts the chemical compositions on the surface of the fabricated 3DPP-1 scaffold by the EDS analysis. Apparently, the Ca element was highly detected in the sample with approximately 59.42% of total weight. Besides, both P and O components were detected in particles around 20.11% and 20.48%, respectively. No other impurity substances were detected in the 3DPP-1 scaffold. A similar result could also be found in the 3DPP-2 and 3DPP-3 scaffolds. This finding indicated the presence of β-TCP inside the fabricated 3DPP scaffolds. Figure 3 portrays the XRD pattern of the investigated 3DPP-1 scaffold. The corresponding spectra indicated that the typical diffraction peaks of β-TCP phase (JCPDS:00-09-0169, Ca_3_(PO_4_)_2_) were detected. In addition, no other precipitate compounds were found in the matrix assuming the formation of a single β-TCP phase in the investigated 3DPP-1 scaffold. The phase identification result could also be detected in the 3DPP-2 and 3DPP-3 scaffolds.

### 3.2. Material Properties of the Investigated 3DPP Scaffolds

**Figure 4:**
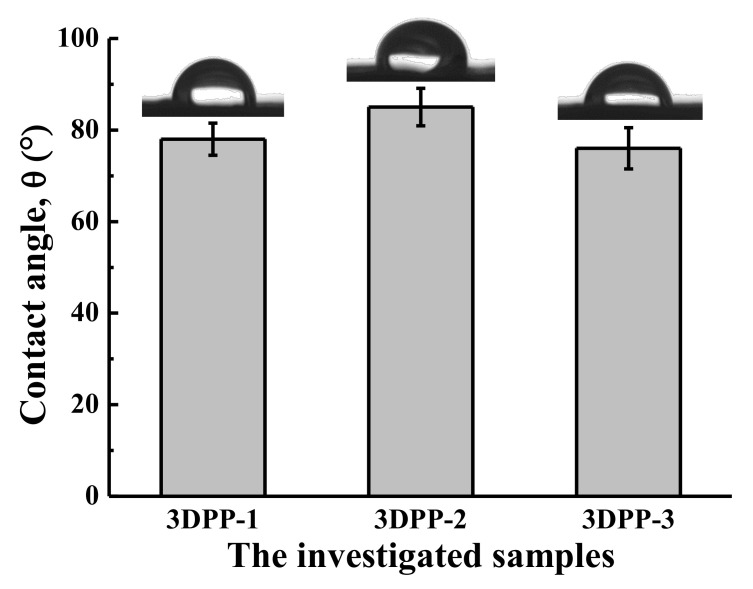
Wettability of the investigated 3DPP scaffolds. The surface is considered hydrophilic when the contact angle is smaller than 90°. No statistically significant difference (*n* = 5) in the hydrophilic contact angle found between all samples tested.

**Figure 5:**
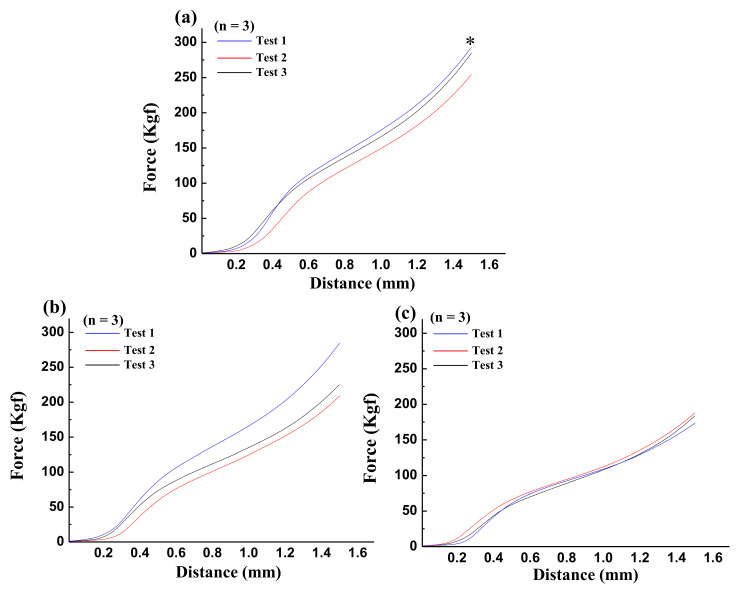
Compression testing results (*n* = 3) of the investigated 3DPP scaffolds: (**a**) 3DPP-1, (**b**) 3DPP-2, and (**c**) 3DPP-3. The 3DPP-1 scaffold exhibited the significantly highest compressive force as compared with 3DPP-2 and 3DPP-3 scaffolds (* *p* < 0.05).

To estimate the cell adhesion ability of the 3DPP scaffolds, wettability testing was performed for the samples. Figure 4 represents the wettability of the investigated 3DPP scaffolds. It was found that the average water contact angles of the 3DPP-1, 3DPP-2, and 3DPP-3 scaffold were less than 90° indicating the hydrophilic feature. However, there is no statistically significant difference in the hydrophilic contact angle found between all samples tested. Figure 5 displays the compression testing results of the investigated 3DPP scaffolds. According to the force versus distance curves, the maximum force of all scaffolds (3DPP-1, 3DPP-2, and 3DPP-3) were measured as 273 ± 20.8 Kgf, 240 ± 39.6 Kgf, and 183 ± 7.6 Kgf, respectively. The 3DPP-1 scaffold exhibited the significantly highest compressive force as compared with 3DPP-2 and 3DPP-3 scaffolds (* *p* < 0.05).

### 3.3. Cell Response and Adhesion Behavior of the Investigated 3DPP Scaffolds

**Figure 6:**
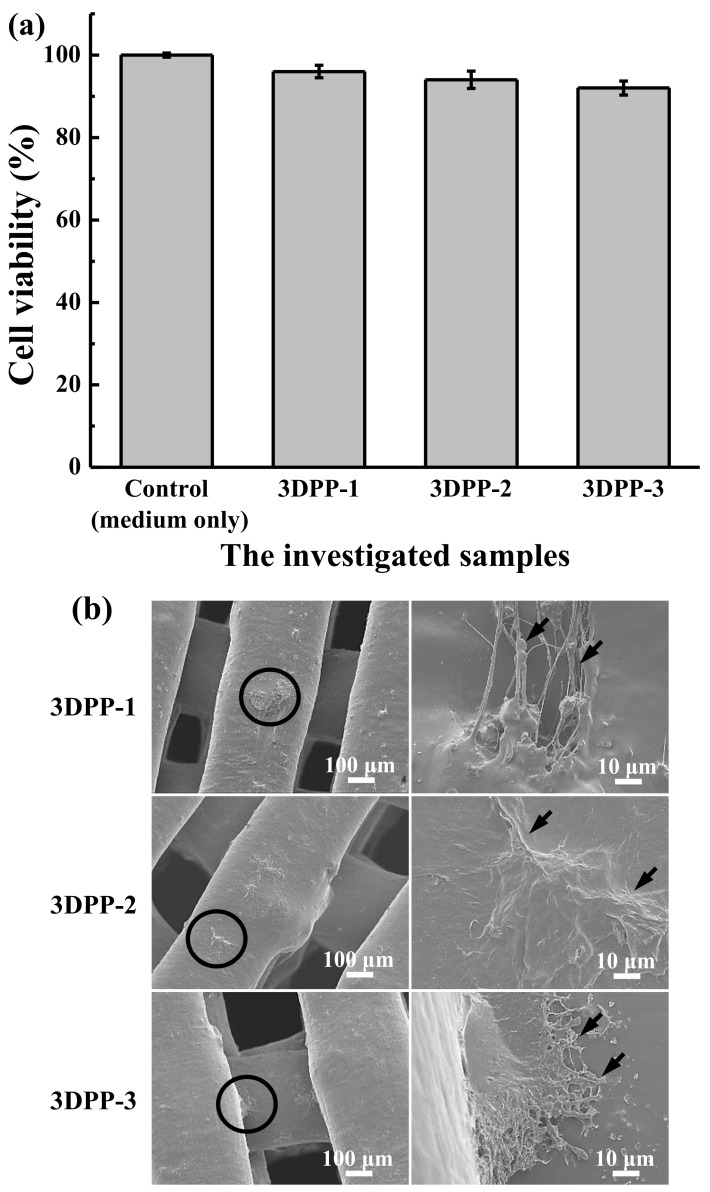
(**a**) Cell viability of MG-63 of the investigated 3DPP scaffolds for 24 h. According to ISO 10993-5 specification, the tested material is considered an acute cytotoxic potential (a short-term culturing experiment (24 h)) if viability value of the tested material is less than 70% of the medium only control (100%). No statistically significant difference (*n* = 5) between tested samples. (**b**) cell morphologies of the investigated 3DPP scaffolds after culturing with MG-63 cells for 3 days. The higher magnification image was taken from the scaffold marked as black circular area. The filopodia (as pointed by arrows) of cells not only adhered flat, but also tightly grabbed the surface structure.

Figure 6a illustrates the cell viability of MG-63 of the investigated 3DPP scaffolds for 24 h. The investigated 3DPP scaffolds exhibited a cell survival rate of more than 70%. According to ISO 109993-5, it is considered an acute cytotoxic potential if the cell viability of the sample is reduced to <70% of the blank. Following cell seeding on the 3DPP scaffolds, morphology and cell adhesion in 3DPP scaffolds were observed via FE-SEM as shown in Figure 6b. After 3 days of cell seeding, it was found that all 3DPP scaffolds showed numerous elongated filopodia. In addition, the filopodia of cells not only adhered flat, but also tightly grabbed the surface structure (as pointed by arrows). The cytotoxic and cell response characteristics demonstrated all 3DPP scaffolds possessed well biocompatibility to osteoblast-like MG-63 cell.

### 3.4. Bone Regeneration of the Investigated 3DPP Scaffolds

**Figure 7:**
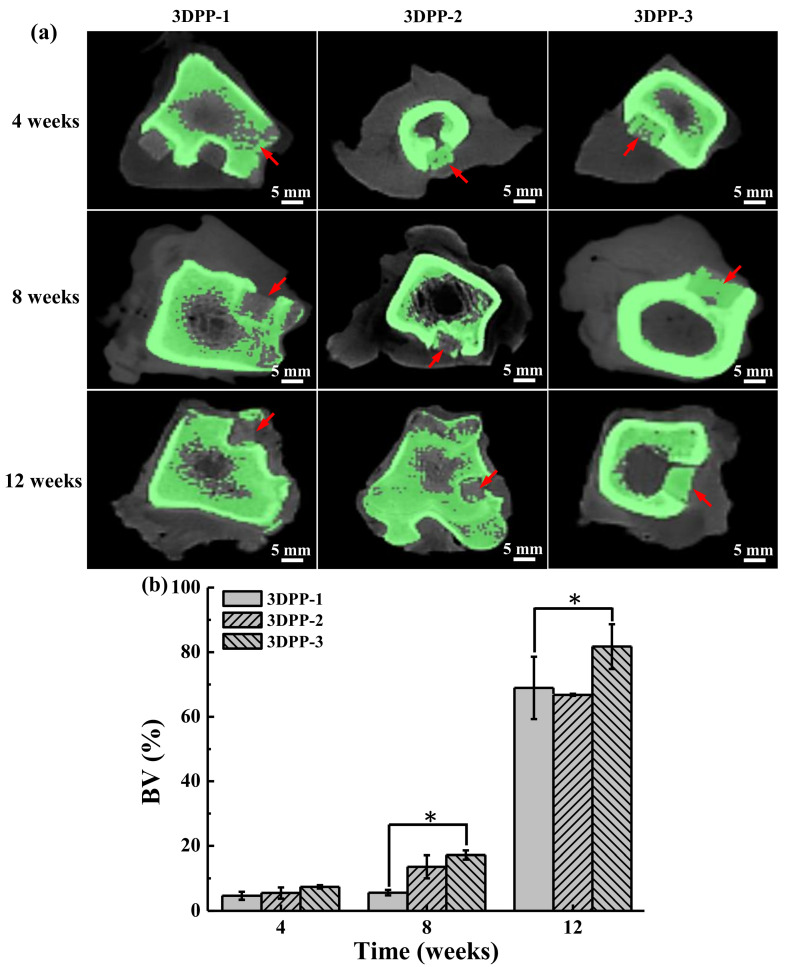
(**a**) Micro-CT images of the 3DPP scaffolds in the lateral condyle of mini-pig after implantation for 4 weeks, 8 weeks, and 12 weeks. Red arrows point the implanted scaffolds; green areas indicate the scanned bone tissues and (**b**) BV in the implanted areas evaluated through micro-CT at week 4, 8, and 12 after implantation. The 3DPP-3 scaffold exhibited significantly difference (*n* = 3) with 3DGP-1 scaffold at week 8 and week 12 (* *p* < 0.05).

Figure 7a highlights the micro-CT images of all 3DPP scaffolds after implantation at serial observational time. At 4 weeks of implantation, apparently, 3DPP-1, 3DPP-2, and 3DPP-3 showed a similar porosity, but after 8 weeks of implantation, it was observed that there is a trend projecting less porosity in the 3DPP-3 than the other two groups. At 12 weeks after the implantation, the 3DPP-3 depicted a dense area similar to the adjacent bone. The micro-CT images demonstrated the newly formed bone mostly occurred in the sites implanted with 3DPP-3. Moreover, the percentage of new bone formation in the implanted area was presented in Figure 7b. It is clearly seen that the percent BV increased in all 3DPP groups as time progressed. No significant difference could be found in the 3DPP groups at week 4. However, the 3DPP-3 scaffold showed a significantly higher percentage of BV than the 3DPP-1 scaffold at week 8 (* *p* < 0.05) and week 12 (* *p* < 0.05), respectively.

## 4. Discussion

In the present study, we fabricated a 3DPP scaffold composed of β-TCP and PF127 that could be beneficial for patients requiring bone implantation. It has been known that a porous designed scaffold can promote bone augmentation and is crucial for cell/tissue conductive and mechanical properties [3,16,24]. In addition, fabricating the scaffold using 3D printing technology enables the production of a customized implantable scaffold with adjustable shape and size according to the defect area [17,25,26]. In this study, a 3DPP scaffold is designed to promote bone tissue ingrowth and loading support for bone defect treatment. We combined the β-TCP and PF127 as these materials possessed both physicochemical and osteoconductive properties. The present study is supported by previous research performed with scaffolds containing tricalcium phosphate (TCP) shown an improvement in bone formation associated with various cell types [25,27]. Similarly, the previous study confirmed that a 3D printed scaffold composed of blended polycaprolactone and bioactive materials such as hydroxyapatite and β-TCP has been successfully used for bone reconstruction [11]. Another study found out that a combination of different bioactive ceramic materials is a promising candidate to generate the 3D-printed scaffold for osteochondral defect reconstruction [13]. 

The modality of 3D printing through the combination of highly ordered scaffold microarchitecture and biomimicry provides control over the structural interval and geometry of scaffold, distribution, and pore size, as well as pore interconnectivity [24]. In this study, these advantages were observed in the FE-SEM images demonstrated intrinsic pores which contributed to the well-interconnected pores of the generated scaffold. A previous study asserted that the interconnected porosity is important to facilitate the exchange of nutrient supply and removal of waste products in the scaffold [24]. Besides, the interconnected porosity will allow the ingrowth of blood vessels into the 3D scaffold [28]. As described in the former study, highly porous microspheres used as bioink in the 3D printed scaffold will support cell adhesion and proliferation before the printing procedure [14]. Accordingly, another study summed up that the β-TCP scaffold with porous architectural characteristics enables cell survival and tissue growth into the scaffold [5,29]. This behavior then profoundly influenced the osteogenesis of post-implantation [5,29]. 

Based on the result of the compression strength analysis, it was found that the ultimate strengths are statistically different between 3DPP-1, 3DPP-2, and 3DPP-3 scaffolds. Compressive strength is a key value for the design of structures. Some materials fracture at their compressive strength limit while others deform irreversibly. Given this fact, the liable amount of deformation may be considered as the limit for the compressive load. In this study, the 3DPP-1 has the highest force of 273 ± 20.8 Kgf compared to other 3DPP scaffolds. Hence, the 3DPP scaffold printed with a pore size of around 200 µm contributed significantly to the improvement of mechanical stiffness. From a mechanical viewpoint, the scaffold should have a strength equal to or even greater than the bone to be repaired [30]. The mechanical properties of the scaffold should match the original bone that can support various external loads, at least during the tissue regeneration process that is ongoing until osseointegration [31,32,33]. The highest average pressure of the 3DPP scaffold (273 ± 20.8 Kgf) will produce a compressive strength of about 24 MPa which resembles human cancellous bone (11–24 MPa), higher than trabecular bone (2–12 MPa) yet much lower than cortical bone (100–250 MPa) [30,34,35]. Although the compressive strength of the 3DPP scaffold is lower than cortical bone. However, Roohani-Esfahani et al. [36] reported that a glass-ceramic scaffold with a compressive strength of 18 MPa is useful to repair large bone defect load bearings. Thus, the 3DPP scaffold has the potential to be a promising candidate as a scaffold to treat large-bone defects in the load-bearing area. Moreover, the 3DPP-1 scaffold has the highest strength because it has the smallest pore size. The smaller the pore size, the greater the mechanical strength of the scaffold because it is denser in structure [23,31,37], while an ideal scaffold should consider a balance between adequate pore size and the required mechanical stability [31]. A previous study assumed that the enhancement of compressive strength from TCP scaffolds influenced by the fabrication method of the TCP powder that will produce the optimal porosity which eventually increased the new bone formation [38]. In this study, the presence of PF127 may affect the microporosity and mechanical properties of the generated scaffolds. Moreover, it is presumed that the biopolymer materials inside the scaffold can act as inorganic phase binders and physical and biological cell colonization modulation when combine with calcium phosphate [29,39]. 

It is generally believed that the normal mammalian cells will always require substrates to adhere and proliferate [40]. The previously published study uncovered that a highly porous micro scaffold provides precise surface areas to allow the cells to attach, penetrate, and grow before the printing procedure [14,41]. Paramount to the success of scaffold should be simultaneously applied for successful organ/tissue regeneration including biocompatibility, space-provision, cell occlusion, and tissue integration [25,26,40]. In the in vitro experiment of this study, the generated 3DPP scaffold highlights a robust cell adhesion on the surface of all substrates and the cells continue to grow on the surface of the materials during the culture period. Hence, this finding indicates that the investigated 3DPP scaffolds possessed good biocompatibility. 

The results of the analysis in this study show that the properties of the 3DPP-3 scaffold are ideal for bone regeneration. This feature can be attributed that pore size scaffolds can influence various factors in bone regeneration. It has been reported that a pore size approaching 300 µm has higher permeability and vascularization potential, which encourages osteogenesis, while some researchers disclose that a pore size between approximately 200 and 350 µm is ideal for osteoblast proliferation [22,23]. Similar to our results with the 3DPP scaffold pore size in the range of 200~350 µm. In general, larger pore sizes are suggested as good candidates for bone regeneration [22,23]. Macropore is ideal for cell ingrowth, while micropore contributes to the increased surface area, triggering ion exchange and adsorption of various proteins [23]. In addition, a larger pore size makes the 3DPP-3 scaffold exhibit a slightly hydrophilic property than the 3DPP-1 and 3DPP-2 scaffolds, which is crucial for the absorption of various biological fluids and proteins, as well as cell attachment [42,43,44,45]. Furthermore, the β-TCP particles were clearly detected in our fabricated 3DPP scaffold verified by the XRD and EDS analysis. This osteoconductive ceramic material contained in the 3DPP scaffold is predicted to promote bone tissue ingrowth when applied in the defect area. Over the years, the β-TCP material has been utilized in bone tissue engineering since its chemical constructs are identical to those of the bone mineralized elements [12,46]. The β-TCP particles have excellent resorbability, bioactivity, and osteoconductivity from the release of Ca and P ions which are vitally important inorganic salts for new bone formation [5,47]. In particular, a previous study confirmed that Ca ions have a desirable effect on cartilage and chondrocyte since it can enhance cell proliferation and differentiation [13]. In light of this, it is probable that Ca and P ions exposed in the 3DPP scaffold would stimulate the formation of bone tissue ingrowth and maintain the structural stability of the scaffold. As discussed above, the 3DPP scaffold with a porous structure (pore size of ~350 μm) is a potential implant to promote the bone cell ingrowth into the porous structure for enhancing bone regeneration. Finally, further studies should be carried out to validate the present findings.

## 5. Conclusions

The microstructural characteristics of the materials verified the pores interconnectivity, hydrophilic feature, and good compressive strength of the 3DPP scaffolds. In addition, in vitro results also demonstrated that the 3DPP scaffolds exhibited non-cytotoxicity and good cell adhesion behavior. The 3DPP scaffold consisting of bioceramic β-TCP and biopolymer PF127 might be simultaneously contributing to stimulating bone tissue ingrowth and providing loading support for bone defect treatment. As a result, the present study suggests that the 3DPP-3 scaffold is estimated as a promising scaffold with great potential to promote bone regeneration for successful implantation in the dental and orthopedic fields.

## Data Availability

Data is contained within the article.
